# Identifying latent comorbidity patterns in adults with perceived cognitive impairment: Network findings from the behavioral risk factor surveillance system

**DOI:** 10.3389/fpubh.2022.981944

**Published:** 2022-09-20

**Authors:** Cristian Ramos-Vera, Jacksaint Saintila, Angel García O'Diana, Yaquelin E. Calizaya-Milla

**Affiliations:** ^1^Research Area, Faculty of Health Sciences, Universidad César Vallejo, Lima, Peru; ^2^Escuela de Medicina Humana, Universidad Señor de Sipán, Chiclayo, Peru; ^3^Escuela de Nutrición Humana, Universidad Peruana Unión, Lima, Peru

**Keywords:** cognitive impairment, multimorbidity, comorbidity, chronic disease, cluster analysis

## Abstract

**Background:**

People with cognitive impairment may be exposed to an increased risk of comorbidities; however, the clustering of comorbidity patterns in these patients is unclear.

**Objective:**

To explore the network structure of chronic comorbidity in a U.S. national sample spanning all 50 U.S. states with more than 170,000 participants reporting perceived cognitive impairment.

**Methods:**

This is a cross-sectional study conducted using Behavioral Risk Factor Surveillance System (BRFSS) secondary data collected in 2019 and covering 49 U.S. states, the District of Columbia, Guam, and the Commonwealth of Puerto Rico. A total of 15,621 non-institutionalized U.S. adult participants who reported “yes” to the subjective cognitive impairment question were considered, of whom 7,045 were men and 8,576 were women. All participants were aged 45 years or older. A statistical graphical model was used that included clustering algorithms and factorization of variables in a multivariate network relationship system [exploratory graphical analysis (EGA)].

**Results:**

The results of the EGA show associations between the comorbid conditions evaluated. These associations favored the clustering of various comorbidity patterns. In fact, three patterns of comorbidities have been identified: (1) arthritis, asthma, respiratory diseases, and depression, (2) obesity, diabetes, blood pressure high, and blood cholesterol high, and (3) heart attack, coronary heart disease, stroke, and kidney disease.

**Conclusion:**

These results suggest the development of interdisciplinary treatment strategies in patients with perceived cognitive impairment, which could help to design an integrated prevention and management of the disease and other related health problems, such as Alzheimer's disease and related dementias.

## Introduction

Cognitive impairment represents one of the greatest public health challenges for health systems and society in general at a global level. This is due to high prevalence rates, which translates into high health care costs, poorer quality of life, and economic and emotional burden for both patients and the medical community and caregivers (1). In the United States, according to a report published in 2019 by the Centers for Disease Control and Prevention, the prevalence of subjective cognitive impairment was 11.1%, equivalent to one in nine adults (2). The prevalence of cognitive impairment in men is 11.3%, compared to women (10.6%) (2). This could be explained by the fact that men generally refuse to use health care services and by the fact that men tend to adopt unhealthy behaviors that affect disease prevention and treatment (3).

Subjective cognitive impairment is defined as the self-reported experience of inability to remember (memory loss), learn new things, concentrate, or make decisions that affect daily life and constitutes one of the earliest noticeable symptoms of Alzheimer's disease and related dementias (ADRD) (1). In fact, some evidence has reported that approximately one in three people who present with cognitive impairment based on clinical diagnoses are likely to develop ADRD within 5 years (4).

Comorbidities, defined as the coexistence of more than one chronic condition in a person, have a high overall prevalence (5). Particularly in the United States, comorbidities represent a public health challenge, constituting a threat to social and economic life in the country (6). Findings from a cross-sectional survey conducted by the National Center for Health Statistics (NCHS) that included 25,417 adults in 2018 reported that 51.8% (129 million) of adults were living with at least 1 of 10 chronic conditions, while 27.2% (68 million) had multiple chronic conditions (6). Comorbidities may be connected through various shared factors, such as genetic, behavioral, or environmental; also, comorbidities may occur because two conditions (physical or mental) have shared or overlapping risk factors, or because one condition results from complications of the other; or because a third condition causes both conditions (5). However, some comorbidities occur fortuitously (5). Therefore, the approach and treatment of comorbidities should focus on why a given pair of disorders tends to coexist.

The relationship between comorbidities and cognitive impairment can be viewed from a bidirectional perspective. On the one hand, people with comorbidities tend to have a higher risk of mental disability, functional impairment, premature death, and poor quality of life (7). In addition, more specifically, comorbidities may lead to an increased risk of cognitive impairment (8). In fact, findings from a meta-analysis of 18 risk factors found that nine were associated with an increased risk of cognitive impairment (9). On the other hand, cognitive impairment could result from various risk factors. These factors can be classified as follows: (a) demographic risk factors, such as advanced age; (b) cardiovascular risk factors, such as diabetes mellitus, dyslipidemia, hypertension in middle age, and obesity in middle age (10); (c) modifiable lifestyle risk factors, such as sedentary lifestyle, smoking, and substance use (10); (c) genetic factors related to the apolipoprotein E (APOE) gene, a risk factor for Alzheimer's disease and related dementias; and psychiatric risk factors such as depression, psychological distress, and sleep disorders (11).

To address and improve the clinical care of patients with cognitive impairment, it is important to identify comorbid conditions. In addition, it will be interesting to uncover whether comorbidities are associated in these patients. Moreover, identifying and understanding the overall network of dependencies that underlie complex multimorbidities in patients with cognitive impairment could provide opportunities for early intervention and development of drugs and other therapies for comorbid conditions and prevention of Alzheimer's disease and other dementias, particularly relevant in an aging society such as the United States. In consideration of this, this study aimed to explore the network structure of chronic comorbidity in a national U.S. sample spanning 49 U.S. states with more than 170,000 participants reporting cognitive impairment.

## Materials and methods

### Data source

A cross-sectional design study was conducted using data from the Behavioral Risk Factor Surveillance System (BRFSS) 2019 survey. For 2019, the state of New Jersey and the U.S. territory of the Virgin Islands were not included because they were unable to collect enough data to meet the minimum inclusion requirements. Furthermore, the other 3 data sets added (i.e., version 1 + version 2 + version 3) were considered. The BRFSS -a major national health-related telephone survey system- is administered and supported by the Centers for Disease Control and Prevention in the United States (12).

The BRFSS collects data on non-institutionalized U.S. adults (≥ 18 years) regarding their health-related risk behaviors, chronic diseases and health conditions, access to health care, and use of preventive services. Data were obtained from the basic component and the optional cognitive impairment module. The basic component contains a set of standard questions that all States use, inquiring about demographic information, health-related perceptions, health-related conditions, and health-related behaviors. The cognitive impairment module targets residents aged 45 years and older and was developed in 2011 to understand the effect and burden of subjective cognitive impairment in the population (12).

Participants who responded to the 2019 BRFSS survey were eligible for inclusion in this study. The inclusion criteria were (a) participants residing in those 32 States that implemented the cognitive impairment model in 2019, (b) residents aged 45 years or older, and (c) those who reported “yes” to the subjective cognitive impairment question. After using the inclusion criteria, 15,621 participants were considered for this study. To arrive at the final sample, the following specifications were considered: 12,367 of the main data set had the variable (CIMEMLOS = yes) from 31 states and the District of Columbia. Two thousand one hundred and eighty-nine from the Version 1 dataset (CIMEMLOS = yes) from 5 states [Iowa, Michigan, Nebraska, Ohio, Utah]. We had to remove the 309 from Iowa because they were included in the main data set. Nine hundred and thirty-five from the version 2 dataset (CIMEMLOS = yes) from 3 states [Kansas, NY, and Oklahoma] and 439 from the version 3 dataset (CIMEMLOS = yes) from 1 state [Maryland].

### Chronic conditions

Data were collected on chronic conditions, for which respondents were asked if they had each of the following conditions: diabetes or high blood sugar, hypertension or high blood pressure, obesity, depression, high blood cholesterol, heart attack, coronary heart disease, stroke, asthma, respiratory diseases, arthritis, and kidney disease. Responses were dichotomized as follows, yes or no.

### Sociodemographic characteristics

Sociodemographic variables in the study included age in years (45–59, 60–69, 70–79, and 80+), education level (≥Bachelor's degree, College, technical school, and ≤High school), employment, income range (< $15,000, $15,000–$50,000, and ≥$50,000), and race (White non-Hispanic, African American, Hispanic, and Other).

### Ethical considerations

The protocol for this study was reviewed and approved by the Research Ethics Committee of the Universidad Peruana Unión. However, the fact that the study included a secondary analysis using an open access database, therefore, the Ethics Committee waived informed consent.

### Statistical analysis

We considered analyzing the psychometric network model called exploratory graph analysis (EGA), through triangulated maximally filtered graph (TMFG), also called EGAtmfg, where the correlations are structurally adjusted (13). This method connects the four variables with the highest partial correlations and then adds up all other variables, one by one, in an iterative manner and runs, from this point, the sum of the highest partial correlations of the three variables whose nodes are included in the network. Through this process, a cluster analysis can be performed, which were detected with the Leiden algorithm, which is a modification of the Louvain algorithm for the detection of communities, with greater speed and accuracy in detecting them (14). In the network, it can be visualized that the strength of the connection represents the strength of the unique association between two variables in the network.

The “EGAnet” package developed by Golino and Epskamp was used (15). The package is available in the R environment (R Core Team, 2019: R Foundation for Statistical Computing, Vienna, Austria; http://www.R-project.org). The network method considers the TMFG procedure with cluster selection using the Leiden/Louvain algorithm mentioned above, which is used as an option in the “EGAnet” package. In performing this analysis, the graphical model, the weights of the edges, and the clusters of the comorbidities were calculated. The weights matrix can be obtained with the “EGA.estimate” function. After this first result, the “bootEGA” function was used to obtain the estimated network structure based on the bootstrap method, under the same parameters with 200 permutations. Both models were calculated under the “cor_auto” argument, structured based on Lavaan's lavCor function (16). In this way, a correlation matrix based on Pearson-type correlations is calculated. This argument eliminates all factors and looks for possible ordinal variables, so ranked if it is ordered or consists of a maximum of 7 unique integer values. Then, an additional check is performed to see if the correlation matrix is positive definite.

After obtaining the bootstrap model, the factor loadings, called standardized node strengths, were calculated using the “net.loads” function. The function “dimensionStability” was used to examine the structural consistency of the predicted network model and the stability of the extracted clusters, to individually evaluate the consistency of the elements in the clusters, the function “itemStability” was used.

Subsequently, we identified which nodes are most important to the network using the node strength centrality metric (the strength with which a node is directly connected to other nodes, based on the absolute sum of edge weights) using the centralityPlot function of the R package qgraph. EGA offers an advantage over traditional factorial methods such as parallel analysis or the Kaiser criterion, especially in models with unbalanced structures, low factor loadings, binary data, and large sample sizes such as those used in the present study (13).

Finally, two entropic fit indexes were analyzed with the “entropyFit” and “tefi” functions: Entropy Fit index with von Neumann Entropy (EFI.vn), Total EFI with von Neumann Entropy (TEFI.vn), in which, in addition to Shannon entropy, quantum information metrics are used, through the correlation matrix (17); such values are more accurate when latent factors are identified. Low values, close to zero, in the EFIs indicate less disorder (or uncertainty) in the system of variables (13).

## Results

The largest proportion (39.8%) of the participants were between 45 and 59 years of age. Female and Non-Hispanic white respondents accounted for 54.9 and 73.9% of the sample, respectively. Slightly more than one third (39.6%) of the participants were unemployed and those reporting household incomes < $15,000 were 20.7%. Only 18.7% were college students. The proportion of participants reporting perceived cognitive impairment and presenting with arthritis, high blood pressure, high blood cholesterol, depression, obesity, and diabetes were 62.6, 61.9, 53.0, 43.1, 36.9, and 27.5%, respectively (see Table 1).

**Table 1:** Sociodemographic characteristics and chronic conditions of the participants of the participants (*n* = 15,621).

**Characteristics**	***N***	**%**
**Age**		
45–59	6,217	39.8
60–69	4,233	27.1
70–79	3,015	19.3
80+	2,156	13.8
**Sex**		
Male	7,045	45.1
Female	8,576	54.9
**Ethnicity**		
Non-Hispanic white	11,544	73.9
Non-Hispanic black	1,875	12.0
Hispanic	1,343	8.6
Other	859	5.5
**Employment status**		
Employed	3,827	24.5
Unemployed	6,186	39.6
Retired	5,608	35.9
**Income**		
<$15,000	3,234	20.7
$15,000– <$50,000	7,873	50.4
≥$50,000	4,514	28.9
**Education**		
Lower school grade	3,343	21.4
High school graduate	5,077	32.5
Some higher education	4,280	27.4
University or higher	2,921	18.7
**Condicion cronic**		
Arthritis	9,775	62.6
Asthma	2,964	19.0
Respiratory diseases	3,603	23.1
Depression	6,739	43.1
Obesity	5,768	36.9
Diabetes	4,302	27.5
High blood pressure	9,681	61.9
High blood cholesterol	8,283	53.0
Heart attack	2,161	13.8
Coronary heart disease	2,573	16.4
Stroke	2,169	13.9
Kidney disease	1,443	9.2

**Figure 1:**
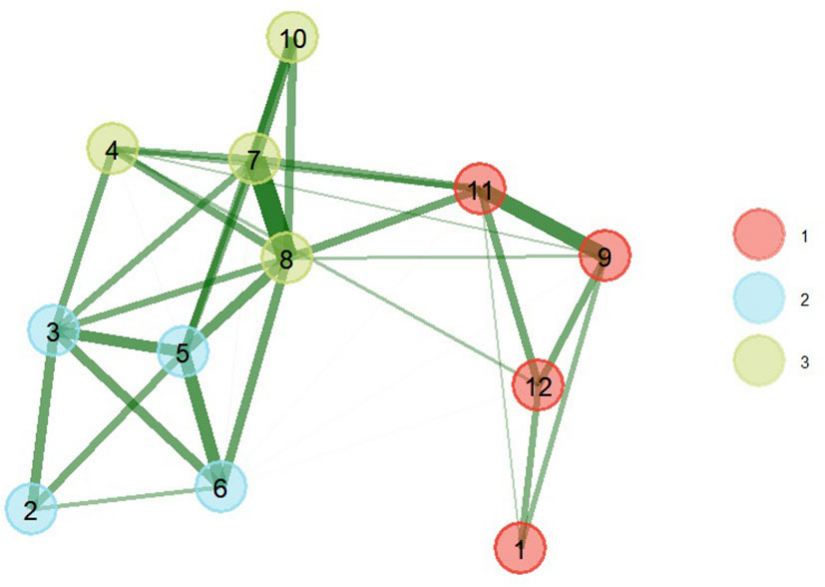
EGA of comorbidity patterns with bootstrap (nboot = 200) (right). The greater the thickness of the connections, the greater the magnitude of the statistical relationships. The thickness of the line is equivalent to the magnitude of the ratio. (1) Arthritis, (2) Obesity, (3) Diabetes, (4) Kidney disease, (5) High blood pressure, (6) High blood cholesterol, (7) Heart attack, (8) Coronary heart disease, (9) Asthma, (10) Stroke, (11) Respiratory diseases, (12) Depression.

The results of the EGA show that the network presents 3 patterns of latent comorbidity, that is to say that chronic conditions are grouped into 3 factors (Figure 1). The first pattern was composed of 4 comorbidities (arthritis, asthma, respiratory diseases, and depression). The second pattern of latent comorbidity included the diagnoses of obesity, diabetes, high blood pressure, and high blood cholesterol. The last factor grouped 4 conditions (heart attack, coronary heart disease, stroke, and kidney disease).

The model obtained after applying 200 parametric bootstrap samples obtained better statistics (better fit) than the base model, which is shown in Figure 1. The triangulated relationships between comorbidities of the same factor of greater magnitude were between heart attack and coronary heart disease (0.69), asthma and respiratory disease (0.49), blood pressure high and blood cholesterol high (0.43), diabetes and high blood pressure (0.41), heart attack and stroke (0.39). The largest associations between comorbidities of different latent groupings were between high blood pressure and coronary heart disease (0.35), high blood pressure and coronary heart disease (0.33), coronary heart disease and respiratory diseases (0.31).

**Figure 2:**
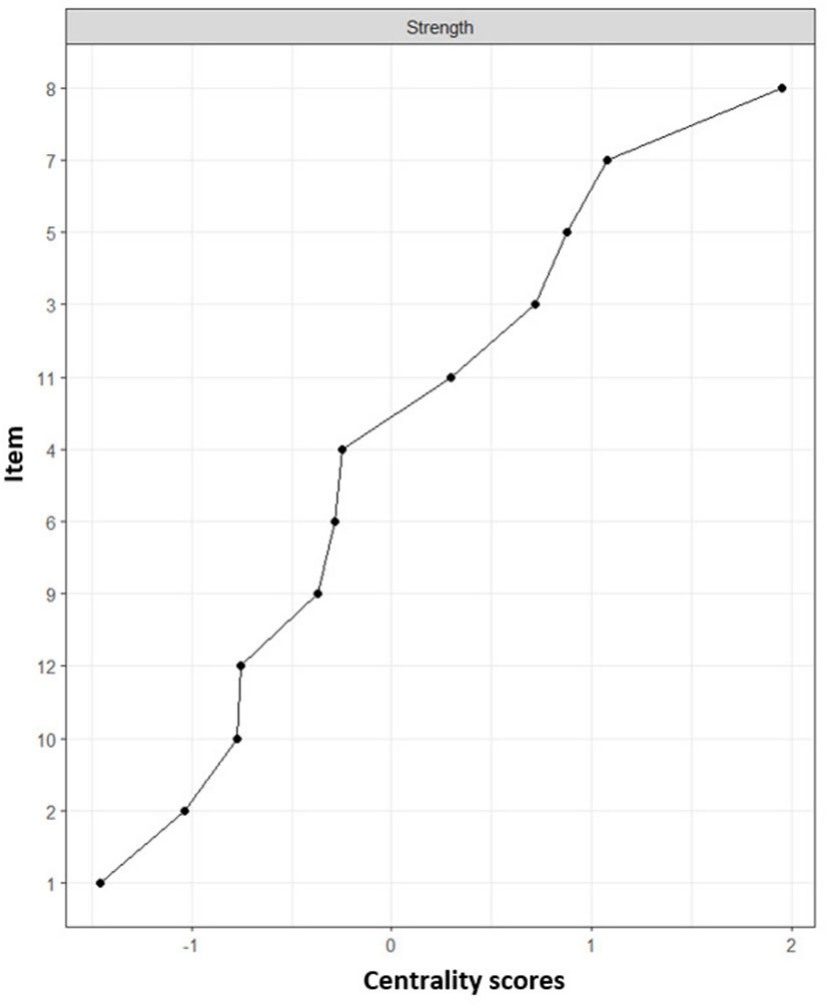
Centrality indexes of chronic conditions. Centrality refers to the measure with the highest number of connections together with the sum of the relationships it presents. Numbers refer to a chronic condition identified in Table 3. (1) Arthritis, (2) Obesity, (3) Diabetes, (4) Kidney disease, (5) High blood pressure, (6) High blood cholesterol, (7) Heart attack, (8) Coronary heart disease, (9) Asthma, (10) Stroke, (11) Respiratory diseases, (12) Depression.

The assignment of items (comorbidities) and network loadings are listed in Table 2. Centrality estimates (Figure 2) in the network show that coronary heart disease (item 8) and heart attack (item 7) were more central in the network, i.e., they presented a greater number and magnitude of connections. While the conditions with lower magnitudes of centrality were obesity (item 1) and arthritis (item 2).

**Table 2:** Grouping of comorbidities according to the EGA.

**Chronic condition**	**Factor**
Arthritis (1)	1
Asthma (9)	1
Respiratory diseases (11)	1
Depression (12)	1
Obesity (2)	2
Diabetes (3)	2
High blood pressure (5)	2
High blood cholesterol (6)	2
Heart attack (7)	3
Coronary heart disease (8)	3
Stroke (10)	3
Kidney disease (4)	3

The EGA refers to 3 patterns of latent comorbidity.

**Figure 3:**
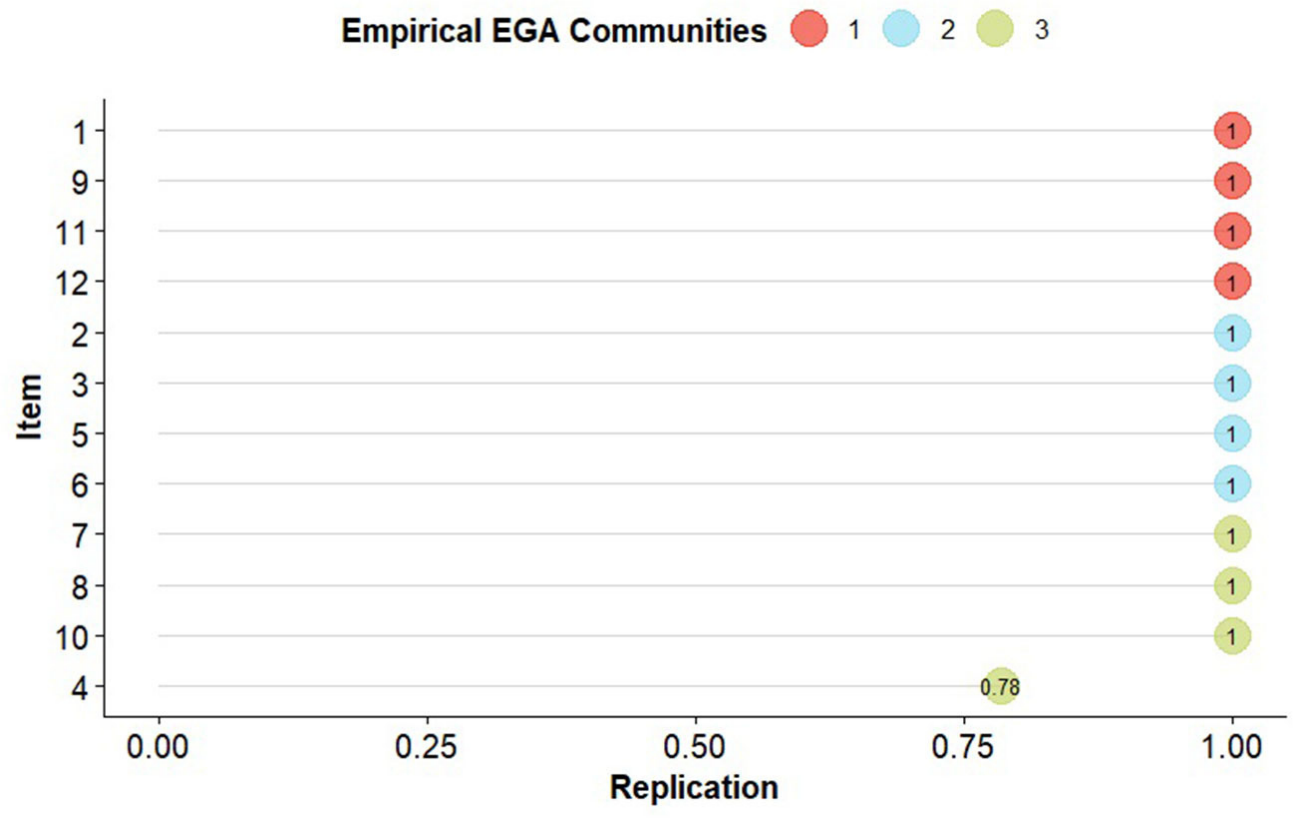
Empirical communities of the 12 chronic conditions of the EGA. The nodes represent each replication of the item (comorbidity) in the original dimension specified by the EGA. (1) Arthritis, (2) Obesity, (3) Diabetes, (4) Kidney disease, (5) High blood pressure, (6) High blood cholesterol, (7) Heart attack, (8) Coronary heart disease, (9) Asthma, (10) Stroke, (11) Respiratory diseases, (12) Depression.

In the result of the clustering using the Louvain algorithm, the 3 identified factors can be observed, with their respective loadings (Table 3) and stabilities (Figure 3) in their grouped dimension, item 4 (kidney disease) can be identified as the most entropic of all (0.78) being below the absolute 1. Regarding the structural consistency of the clusters, these obtained values of 1, except for the third cluster, which obtained (0.79), while, in the general stability, cluster 1 obtained a lower value (0.95). Concerning the entropic adjustment indexes, EFI = −1.21; TEFI = −7.15, being close to 0, these can be considered acceptable for the model found.

**Table 3:** Loads of chronic conditions in clusters in the EGA.

	**Cluster 1**	**Cluster 3**	**Cluster 2**
Arthritis (1)	**0.231**	0	0
Asthma (9)	**0.461**	0	0.082
Respiratory diseases (11)	**0.429**	0	0.274
Depression (12)	**0.394**	0	0.040
Obesity (2)	0	**0.313**	0
Diabetes (3)	0.027	**0.439**	0.295
High blood cholesterol (5)	0	**0.458**	0.344
High blood cholesterol (6)	0	**0.377**	0.128
Heart attack (7)	0.141	0.227	**0.491**
Coronary heart disease (8)	0.241	0.394	**0.470**
Stroke (10)	0	0.102	**0.264**
Kidney disease (4)	0.198	0.123	**0.212**

Bold represent the highest loadings on each factor identified in the EGA.

Upon inspection of the network loadings (Table 3), items 7 (heart attack), 8 (coronary heart disease), and 9 (asthma) report higher factorial complexity since they report higher factor loadings in two dimensions. In addition, coronary heart disease, heart attack, and kidney disease have loadings >10 on all three comorbidity dimensions. It should be noted that network loadings tend to be lower than factor loadings since it uses a covariance of partial relationships, therefore low values should not be interpreted as weak loadings.

With respect to the structural consistency of the clusters (see Table 4), these obtained values of 1, except for the third cluster, which obtained (0.79), while, in the general stability, cluster 1 obtained a lower value (0.95). Finally, as for the entropic adjustment indexes, EFI.vn = −1.21; TEFI.vn = −7.15, being close to 0 can be considered acceptable for the model found.

**Table 4:** Internal consistency of the EGA.

**Cluster**	**Cluster structural**	**Average item**
	**consistency**	**stability**
1	1	0.95
2	1	1
3	0.79	1

## Discussion

The findings of this study support the evidence that comorbidities are the norm rather than the exception among persons with perceived cognitive impairment. The presence of comorbidities may lead to an increased risk of cognitive impairment (8). There is a need to address and improve the clinical care of patients reporting perceived cognitive impairment, taking into account the identification of comorbid conditions, as well as the relationships between them, because this could provide opportunities for early intervention, development of drugs and other therapies for comorbid conditions, and prevention of Alzheimer's disease and other dementias particularly relevant in U.S. society. Therefore, this study aimed to explore the network structure of chronic comorbidity in a US national sample (BRFSS) that included participants who reported perceived cognitive impairment.

In the present study, we have evidenced three patterns of comorbidities in patients with perceived cognitive impairment. The first pattern consisted of chronic conditions, such as arthritis, asthma, respiratory diseases, and depression. Cross-sectional studies that conducted factor analyses and multivariate relationship network models in the United States and Europe reported clusters that include these chronic conditions (18, 19). The second comorbidity pattern integrated obesity, diabetes, high blood pressure, and high blood cholesterol. Similarly, in a study in which exploratory factor analysis was used to identify clusters of multimorbid chronic conditions that do not occur at random, similar patterns have been identified (20). The latter pattern included diseases, such as heart attack, coronary heart disease, stroke, and kidney disease. The latter pattern has been reported more frequently in several similar studies (21, 22).

In addition, shared latent network loadings of coronary heart disease (higher network centrality) with the first two dimensions of comorbidity have been reported, which may indicate an underlying link between such conditions and their relationship with cognitive impairment. While diabetes, hypertension, and respiratory diseases presented cross-network loadings with the third latent factor [cardiovascular (CVD) and renal diseases]. Another investigation that considered older adults with neurological problems in the United Kingdom reported a cardiovascular comorbidity group with a higher prevalence that included such conditions (23). In addition, another investigation conducted in the Chinese population using the factor analysis method to group comorbidity patterns reported that diabetes and hypertension share a common underlying cause with CVD and cerebrovascular disease (24), such non-communicable diseases guide certain comorbidity pathways in the systemic representation of multimorbidity in American adults with perceived cognitive impairment.

### Pattern 1: Arthritis, asthma, respiratory diseases, and depression

Arthritis is a chronic autoimmune inflammatory disease that leads to progressive joint damage and is associated with vascular, metabolic, and psychological comorbidities (25). Arthritis is considered to represent a major global public health challenge, as its overall prevalence and incidence rates are increasing worldwide at a rapid pace (25). Pulmonary conditions (asthma and other respiratory diseases) are common in patients with arthritis and affect all components of the lung (26). This could be due to several risk factors, such as older age, male sex, tobacco use, and elevated levels of antibodies to citrullinated peptide antigens (ACPA) (27). In fact, it has been evidenced that tobacco exposure is capable of promoting citrullination of proteins at the lung level (28). It is suggested that blood ACPA levels are highly associated with arthritis in patients with lung disease (26).

On the other hand, the coexistence between arthritis and depression is evident. In fact, it has been reported that 13–20% of patients with rheumatoid arthritis may present comorbid depressive symptoms (29). Also, two recent large studies in the U.S. population have reported an increased risk of depression among patients with rheumatoid arthritis, osteoarthritis, systemic lupus erythematosus, gout, and psoriatic arthritis, compared to those without these conditions (30, 31). Arthritis patients may be a high-risk group requiring special attention for the prevention and management of depression.

Additionally, this first pattern included pulmonary conditions and depression. This association has been described in several studies in which the impact of depression on respiratory diseases, including asthma and COPD, was established (32). These chronic conditions are highly prevalent worldwide and in the U.S. population in particular, representing a social and economic burden on the public health system (33). Depressive symptomatology is prevalent in patients diagnosed with respiratory diseases and represents a challenge for treatment and management (34). In addition, the presence of depression in patients with respiratory disease is associated with higher rates of mortality, disease exacerbation, length of hospital stay, and lower quality of life and functional status (35). One study showed that 43% of the increased risk of developing asthma among adults was due to the presence of depression (36). The results of a cross-sectional survey of 18,588 adults in the United States showed that depression was common among those with COPD; furthermore, they reported that it is more likely to occur in COPD than in other common chronic diseases (37).

In the current study, more than half (62.6%) of the participants were found to have arthritis. Arthritis may go along with cognitive impairment (38). Findings from a study of U.S. subjects showed that the proportion of participants with arthritis who presented with cognitive impairment ranged from 8 to 29% (39). The pathophysiological pathways of these pathologies in cognitive impairment are complex and could be explained by common risk factors, response to symptomatology, and biochemical alterations. For example, arthritis, as a chronic inflammatory disease, can increase the expression of major proinflammatory cytokines, such as tumor necrosis factor-α, interleukin-6 and interleukin-1β, which, through the brain, affect pathophysiological and neurotransmitter functions relevant to depression (40). Moreover, the pain and fatigue common in arthritis can cause and worsen depression (41). On the other hand, some mechanisms underlying the association between asthma and respiratory diseases with cognitive impairment have been suggested. Prominent among these mechanisms are the fact that asthma causes aberrations of synaptic tissues and blood vessels, elevated levels of hypoxia-inducible factor-1alpha (HIF-1α) and hypoxia-induced factor-2α (HIF-2α) (42). Based on this phenomenon, it is suggested that asthma may cause oxygen deprivation and eventually lead to cognitive impairment (42). These findings imply that, during clinical evaluations of cognitive impairment, patients with this pathology should also be evaluated to detect diseases, such as arthritis, asthma, respiratory diseases, and depression; although it is not a standard practice in the clinical evaluation of perceived cognitive impairment.

### Pattern 2: Obesity, diabetes, high blood pressure, and high blood cholesterol

The association of these comorbidities has been known for several decades and they have been defined as “metabolic syndrome” since the 1980s (43). Despite the lack of consensus on the definition, the metabolic syndrome is considered a set of risk factors, such as hypertension, central obesity, insulin resistance (a precursor of diabetes), and atherogenic dyslipidemia, which together increase the probability of presenting several chronic conditions, such as atherosclerotic cardiovascular disease, type 2 diabetes mellitus, and vascular and neurological complications, such as stroke (44). Metabolic syndrome, as a cardio metabolic risk factor, is highly prevalent among adult populations and has high costs for public health systems worldwide, particularly in the United States (44).

The origin of the metabolic syndrome is complex and multifaceted, and has not yet been fully elucidated; however, it has been suggested that visceral adiposity represents a major trigger for most of the pathophysiological pathways involved in the metabolic syndrome, which confirms the implication of high caloric intake as a major causal factor (45). Furthermore, it is important to mention that all proposed mechanistic factors, such as insulin resistance, neurohormonal activation, and chronic inflammation appear to be major contributors to the onset and progression of the metabolic syndrome and its subsequent transition to CVD and type 2 diabetes mellitus (46). The importance of environmental and lifestyle factors, such as excessive consumption of high-calorie dense foods and lack of regular physical activity, has also been emphasized and may play a fundamental role in its development (46, 47). Therefore, lifestyle improvements, particularly in physical activity and the adoption of dietary patterns that include fruits and vegetables, legumes, nuts, whole grains, protein, and high quality fats from fish and seafood, as well as a limited intake of refined carbohydrates, sodium, and saturated fatty acids can be considered as one of the main therapeutic strategies for the prevention, treatment, and management of metabolic syndrome.

On the other hand, it was shown that cognitive impairment is also associated with metabolic syndrome (48). In fact, CVD and cerebrovascular disease may share underlying mechanisms and risk factors (49). This could be explained by the fact that chronic metabolic damage may contribute to the appreciation of atherosclerosis in the small blood vessels of the brain, which results in white matter damage and cognitive dysfunction. One of the main causes of cognitive impairment is the permanence of cognitive deficits and dementia, also known as “vascular cognitive impairment” due to cerebrovascular disease (50). In addition, substantial evidence has suggested that vascular risk factors may lead to sporadic Alzheimer's disease (50). Therefore, beyond the direct negative impact of the metabolic syndrome on the vascular system, increasing the risk of major cardiovascular events and type 2 diabetes mellitus, there appears to be an underlying pathophysiological association with neurodegenerative disorders, particularly cognitive impairment (48).

### Pattern 3: Heart attack, coronary heart disease, stroke, and kidney disease

CVD includes a number of cardiac conditions including (a) coronary heart disease, either angina or heart attack, (b) stroke, which is caused by a blockage of a blood clot (known as ischemic stroke) or the rupture of a blood vessel and bleeding (known as hemorrhagic stroke), and (c) peripheral vascular disease which refers to the obstruction of the large blood vessels that supply blood to the arms and legs (51).

Inflammation, insulin resistance, lipids and fatty acids, hemostasis/fibrinolysis, oxidative damage, and stress, and the role of endothelial cells, among other factors have been proposed as key pathways in the initial development and progression of CVD (52). Most of these pathophysiological pathways are important not only in CVD, but also in other chronic inflammatory diseases, such as type 2 diabetes mellitus, renal disease, and respiratory diseases (52). Therefore, these common pathways are of particular interest in examining the full spectrum of these chronic diseases, from onset through progression to clinical stages. They can also help define the potential elevated risk for heart disease in patients with other inflammatory pathologies, as biomarkers are a powerful tool to diagnose, detect, or provide prognostic information for pathological conditions (53).

On the other hand, such CVD is also closely related to an unhealthy lifestyle and a variety of modifiable behaviors, such as tobacco use, sleep duration, physical activity, and diet (54). Results from a large U.S. cohort have demonstrated additional benefits of maintaining healthy lifestyle behaviors to decrease the risk of CVD (55). Similarly, there is growing evidence to suggest that spending more time sitting during work and leisure time, as well as having inadequate sleep (spending too much or too little time sleeping), are also important predictors of all-cause mortality and CVD (56). Therefore, healthy behaviors including being physically active, not smoking, and maintaining a healthy weight, getting adequate rest, and eating a healthy diet are important in achieving CVD prevention (54).

Likewise, this pattern evidenced the clustering of CVD with kidney disease. Heart disease is a risk factor for kidney disease (57). Moreover, the relationship between the two chronic conditions is bidirectional, as kidney disease can also cause CVD (52, 57). In particular, remodeling of the myocardium and blood vessels can lead to various cardiovascular complications, such as cardiomyopathy, atherosclerosis, arterial stiffness, calcification and subsequent ischemic heart disease, heart failure, cerebrovascular and cardiovascular death in patients with chronic kidney disease (58). On the other hand, CVD, especially those involving a systemic inflammatory process, such as atherosclerosis, continue to be the main cause of morbidity and mortality in patients with chronic kidney disease. Furthermore, heart disease is one of the most common causes of death among people undergoing dialysis (59). Several theories have been proposed to explain the implications of CVD in kidney disease, including the fact that kidney disease itself represents an independent risk factor for CVD (60). In addition, it is associated with higher rates of traditional and non-traditional CVD risk factors (60). As well, factors such as estimated glomerular filtration rate, albuminuria, and even microalbuminuria without a decrease in renal function, are associated with an increased prevalence of CVD risk (59).

CVD, especially coronary heart disease, represents one of the main modifiable risk factors for dementia or cognitive impairment (61). Substantial evidence shows a relationship between other types of CVD, such as atrial fibrillation with cognitive impairment or risk of dementia (8). In fact, the results of a meta-analysis of 7 prospective studies reported that people with atrial fibrillation had a 36% higher risk of developing cognitive impairment or dementia (62).

### Clinical and public health implications

The present study has improved and broadened our understanding of the fact that diseases are associated with each other and the importance of not considering them as a series of isolated conditions. The findings of this research can be used to guide physicians, in particular, and health professionals, in general, in determining and assessing comorbid risk in patients with cognitive impairment, prioritizing a general intervention and prevention strategy at the individual level. In addition, the study can be used to favor the formulation and implementation of policies and strategies to identify people with cognitive impairment at high risk of metabolic syndrome, CVD, to favor timely interventions focusing on shared modifiable risk factors, such as obesity, hypercholesterolemia, hypertension, smoking, physical inactivity, inadequate dietary patterns, sleep duration, among others.

### Limitations

Our study has some limitations. This is a study with a cross-sectional approach that does not allow causal inference of the associations found between chronic conditions with comorbidities patterns; therefore, interpretation of the findings should be made with concern. On the other hand, all diseases were self-reported, it is possible that the data may not be accurate due to recall bias; therefore, future research should be based on clinical cognitive diagnoses or medical record analysis of cognitive impairment and comorbid conditions performed by a specialist physician based on medical care to minimize bias. However, despite these limitations, we believe that the findings broaden and provide insight into the combination of comorbid conditions, because the type and number of chronic conditions have not been limited as a criterion for grouping patterns.

## Conclusion

In the present cross-sectional study, associations between chronic conditions were found to exist. Such associations favored the clustering of various comorbidity patterns. In fact, three patterns of comorbidities have been identified: (1) arthritis, asthma, respiratory diseases, and depression, (2) obesity, diabetes, blood pressure high, and blood cholesterol high, and (3) heart attack, coronary heart disease, stroke, and kidney disease. Based on these results, the development of interdisciplinary treatment strategies in patients with perceived cognitive impairment is suggested, which could help to design an integrated prevention and management of the disease and other related health problems such as Alzheimer's disease and related dementias.

## Data availability statement

Publicly available datasets were analyzed in this study. This data can be found at: https://www.cdc.gov/brfss/data_documentation/index.htm.

## Ethics statement

The studies involving human participants were reviewed and approved by Research Ethics Committee of the Universidad Peruana Unión. The patients/participants provided their written informed consent to participate in this study.

## Author contributions

CR-V, JS, and AO'D contributed to the conceptualization and formal analysis of the manuscript. JS and YC-M assisted in data curation and preparation of the original draft. All authors have read and accepted the published version of the manuscript. All authors contributed to the article and approved the submitted version.

## Conflict of interest

The authors declare that the research was conducted in the absence of any commercial or financial relationships that could be construed as a potential conflict of interest.

## Publisher's note

All claims expressed in this article are solely those of the authors and do not necessarily represent those of their affiliated organizations, or those of the publisher, the editors and the reviewers. Any product that may be evaluated in this article, or claim that may be made by its manufacturer, is not guaranteed or endorsed by the publisher.
